# The Future Is Coming: Artificial Intelligence in the Treatment of Infertility Could Improve Assisted Reproduction Outcomes—The Value of Regulatory Frameworks

**DOI:** 10.3390/diagnostics12122979

**Published:** 2022-11-28

**Authors:** Sanja Medenica, Dusan Zivanovic, Ljubica Batkoska, Susanna Marinelli, Giuseppe Basile, Antonio Perino, Gaspare Cucinella, Giuseppe Gullo, Simona Zaami

**Affiliations:** 1 Department of Endocrinology, Internal Medicine Clinic, Clinical Center of Montenegro, School of Medicine, University of Montenegro, 81000 Podgorica, Montenegro; 2 Clinic of Endocrinology, Diabetes and Metabolic Disorders, University Clinical Center of Serbia, 11000 Belgrade, Serbia; 3 Medical Faculty, Ss. Cyril and Methodius University of Skopje, 1000 Skopje, North Macedonia; 4 School of Law, Polytecnic University of Marche, 60121 Ancona, Italy; 5 IRCCS Orthopedic Institute Galeazzi, 20161 Milan, Italy; 6 Department of Obstetrics and Gynecology, Villa Sofia Cervello Hospital, IVF UNIT, University of Palermo, 90146 Palermo, Italy; 7 Department of Anatomical, Histological, Forensic and Orthopedic Sciences, “Sapienza” University of Rome, 00161 Rome, Italy

**Keywords:** artificial intelligence (AI), infertility, assisted reproductive technology, oocyte, embryo, legal and regulatory frameworks

## Abstract

Infertility is a global health issue affecting women and men of reproductive age with increasing incidence worldwide, in part due to greater awareness and better diagnosis. Assisted reproduction technologies (ART) are considered the ultimate step in the treatment of infertility. Recently, artificial intelligence (AI) has been progressively used in the many fields of medicine, integrating knowledge and computer science through machine learning algorithms. AI has the potential to improve infertility diagnosis and ART outcomes estimated as pregnancy and/or live birth rate, especially with recurrent ART failure. A broad-ranging review has been conducted, focusing on clinical AI applications up until September 2022, which could be estimated in terms of possible applications, such as ultrasound monitoring of folliculogenesis, endometrial receptivity, embryo selection based on quality and viability, and prediction of post implantation embryo development, in order to eliminate potential contributing risk factors. Oocyte morphology assessment is highly relevant in terms of successful fertilization rate, as well as during oocyte freezing for fertility preservation, and substantially valuable in oocyte donation cycles. AI has great implications in the assessment of male infertility, with computerised semen analysis systems already in use and a broad spectrum of possible AI-based applications in environmental and lifestyle evaluation to predict semen quality. In addition, considerable progress has been made in terms of harnessing AI in cases of idiopathic infertility, to improve the stratification of infertile/fertile couples based on their biological and clinical signatures. With AI as a very powerful tool of the future, our review is meant to summarise current AI applications and investigations in contemporary reproduction medicine, mainly focusing on the nonsurgical aspects of it; in addition, the authors have briefly explored the frames of reference and guiding principles for the definition and implementation of legal, regulatory, and ethical standards for AI in healthcare.

## 1. Introduction

Infertility is a global health issue of women and men of reproductive age with increasing incidence worldwide, in part due to improved awareness and better diagnosis. It is defined as the failure to achieve pregnancy after 12 months of regular unprotected sexual intercourse [1]. Besides female and male infertility causes, an unknown cause is present in circa 85% infertile couples, 15% of whom have unexplained infertility [2]. Female infertility causes include: ovarian dysfunction and anovulation, tubal infertility, endometriosis, and diminished ovarian reserve. A male factor is likely to be a primary or contributing cause in approximately 50% of couples and can be related to congenital, acquired, or idiopathic factors that impair spermatogenesis [3]. The best approach to initiate a diagnostic–therapeutic pathway is the simultaneous evaluation and treatment of both female and male infertility factors [4]. Infertility treatment includes ovulation induction and/or stimulation in order to produce multiple mature ovarian follicles. Both clomiphene citrate, a selective estrogen receptor modifier, and letrozole, an aromatase blocker, cause an increase of hypothalamic gonadotropin-releasing hormone (GnRH) pulse frequency and pituitary gonadotropin secretion-inducing ovarian folliculogenesis [2]. The main problem is a multiple pregnancy rate of less than 10%, most of which are twin gestations, and the risk of ovarian hyperstimulation syndrome [5,6].

In women with hypogonadotropic hypogonadism, pulsatile GnRH administration induces follicular maturation and ovulation due to the stimulation of endogenous gonadotropins. Folliculometry and planned intercourse or intrauterine insemination (IUI) may be used to achieve fertilization at the time of ovulation [2]. IUI is a first-line treatment for mild male fertility, and it could be combined with ovarian stimulation in the treatment of couples. If not successful, in vitro fertilization (IVF) is the next step. Undergoing an IVF program consists of a GnRH agonist or antagonist protocol, individually chosen, followed by gonadotropin stimulation. The initial doses of gonadotropin are adjusted according to patient age, estimated ovarian reserve, and response to prior stimulation, and are set during the first three to four days. Transvaginal ultrasonography and measuring of blood estradiol [E2] are used to estimate the ovarian response in order to modify the gonadotropin dose, and final oocyte maturation is induced [7].

In further courses, oocyte retrieval is carried out, and those chosen based on quality undergo IVF or, in the case of male factor infertility, intracytoplasmic sperm injection (ICSI) [8]. ICSI is also recognised as a method of choice for women with thyroid autoimmunity (TAI) undergoing ART [9,10]. Embryos are cultured under optimal conditions [11], and then those of the highest quality are transferred on the second, third, or fifth day after oocyte aspiration into the uterus under ultrasound guidance (US is not mandatory) [2]. Over the next 5–10 years, further increases in birth rates in women with infertility are expected due to greater awareness of lifestyle factors, as well as a possible refinement of current ART and the development of new forms of treatment relying on germ cell manipulation, artificial gametes, genetic screening of embryos, and gene editing of embryos [12]. Donor oocytes or sperm may be options in specific situations.

### Artificial Intelligence in Reproductive Medicine

Artificial intelligence (AI) as an official term first appeared at the Dartmouth conference in 1956. Ever since, it has been used as a reference to the continual study of artificial intelligence. Such a wide-ranging research endeavour hinges on the use of devices such as computers to reproduce human mental processes, such as cognition, learning, decision making, judging, and language usage [13]. Nowadays, AI is already in use in a lot of different industries, and there is a lot of ongoing research. There are a lot of fascinating applications of AI, such as in the transport and automotive industry (e.g., development of autonomous vehicles [14], transport mapping [15], solving financial problems [16], face and speech recognition [17,18]), and others which raise concerns and are potentially destructive, such as the development of Lethal Autonomous Weapon Systems (LAWS) [19], e.g., missile systems with selective targeting capabilities and learning machines with the cognitive ability to select their enemies with no need for intervention by humans [20]. The developmental foundations for this research area are grounded in a subdiscipline of philosophy called philosophy of mind. Assumptions about the mind such as connectivism, computation theory of mind, and behaviourism are essential for understanding AI [21].

In order to optimally run software needed for AI, adequate hardware is essential. For example, to run some deep learning algorithms, graphics processing units (GPUs) produced by “Nvidia” are used. In modern times, the amount of electronic data created every day is astonishing. Technological advancements have been made for storing this data. This was a necessity to accommodate the 2.5 quintillion bytes saved each day [22,23]. Such an amount of data needs some kind of analytics that are automated; hence, AI is a very powerful tool to fulfil that task. The core element that constitutes AI is algorithms. In general, algorithms are specific steps to solve some problem. For a computer to be able to “read “and process vast amounts of data, a specific type of technology is needed, so-called natural language processing (NLP). NLP uses algorithms that allow computers to “understand“ and process human language, so this part of AI research is tightly connected with linguistics. This technology is used to construct information that is valuable and meaningful from some unstructured data, such as electronic medical records. In that way, a computer is able to further analyse data given to it [24]. The cornerstone of AI is constituted by empirical machine learning algorithms. There are different types of machine learning, and they are classified by how they analyse data and their level of dependence.

In short, machine learning (ML) is classified in three main groups: ML capable of recognizing patterns (unsupervised ML), ML that has algorithms that perform classification and prediction based on previous examples (supervised ML), and ML that uses a system with reward and punishment methods to form a solution strategy to solve some problem (reinforcement learning) [25]. The first big success of AI in medicine started with predictions of protein complexes in molecular medicine that led to some new drug targets discoveries. The big data that electronic medical records (EMRs) and hospital data have within are perfect for AI to analyse and give some useful information. The current status of EMRs is that they are cumbersome and lack inter-record communication. It is of great value to analyse this vast amount of data. An example includes AI that has the ability to detect subjects that are at risk for chronic disease; furthermore, AI could facilitate faster, more efficient health system calculations of cost–benefit ratios and help in decision making. AI is vastly used nowadays to analyse and learn to recognise patterns in image processing, so there is great interest in fields such as radiology, pathology, ophthalmology, and dermatology—so-called “vision- orientated specialties“. It is of great value that AI can reduce common errors in everyday clinical practice and make predictions in real-time [26,27].

In reproductive medicine (RM), AI application started in the late twentieth century. Nowadays, there are a lot of different subtypes of AI technology that have applications in reproductive medicine. Supervised learning methods (decision tree, support vector machines and naive Bayes classifier) are mostly used in non-surgical areas of RM. These algorithms need human assistance and use instances supplied externally to predict the fate of instances given in the future; they are designed to categorise data from given information [28,29]. So, the usage of this type of AI found its application in determining the morphokinetic parameters of the embryos that are most optimal [30], determining cost effectiveness in human oocyte cryopreservation [31], predicting IVF and ICSI outcomes, and classification of sperm cells [32]. Unsupervised learning models are not yet fully used in RM. Such algorithms are effective at so-called class discovery, since they use unlabeled data to “discover” the underlying structure and relationship within and to cluster it [33]. Types of unsupervised ML used are principal component analysis and K means that are mostly used in image processing. They can predict pregnancy based on the quality of oocytes, with a success rate of around 60% [34]. Other subtypes of AI commonly used in medicine include artificial neural networks (ANN). ANNs borrow structures from neuronal connections and consist of layers such as the input layer (where data start to be analysed), inner or hidden layer (where data are analysed), and output layer (where final data are presented), with weight connections and bias nodes between these layers. Deep networks are complex forms of ANN, and these AI technologies are widely used today in speech recognition, visual object recognition, and object detection, as well as in medical fields, such as drug discovery and genomics [35]. In RM, ANN are used in embryo segmentation [36], to describe blastocyst expansion and rank-order blastocysts for transfer [37], and to predict an overall outcome of IVF.

Meanwhile, for robotic aspects of RM, reinforcement AI machine learning is more commonly used [28,38]. Robotic surgery serves as a bridge between conventional open surgery and the minimally invasive laparoscopic surgery, and there are currently several different applications in this field. Examples are robotics-assisted myomectomy, tubal reanastomosis, endometriosis, ovarian tissue cryopreservation, and ovarian transposition. In addition, their applications include male infertility operations such as vasectomy reversal, subinguinal varicocelectomy, targeted spermatic cord denervation, and robotics-assisted microsurgical testicular sperm extraction (microTESE). These procedures, although costly and time consuming, have good results in terms of shorter hospital stay, decreased blood loss, less post-operative pain, and faster convalescence compared to open or laparoscopic surgeries, whereas reproductive outcomes were described as similar to nonrobotic surgical approaches [38,39,40].

Overall, AI has the ability to do work that would require thinking with strict or not-so-strict guidelines given by humans, with AI being able to analyse pixels from pictures and videos and recognise the context of the given image, as well as analysing text information and predicting outcome based on the given inputs.

**Figure 1:**
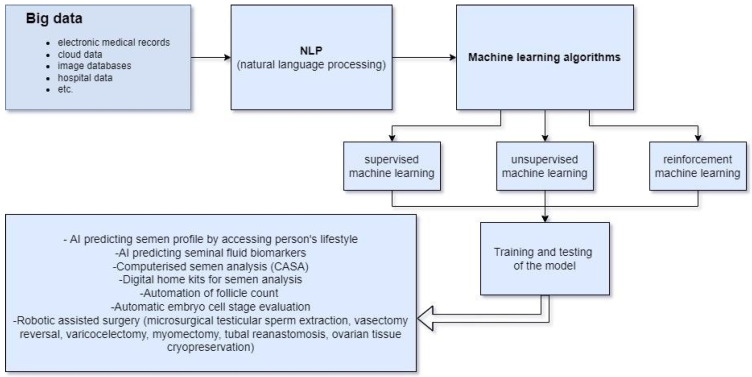
Usage of AI in reproductive medicine.

Most of the research done in AI applied to RM is still lacking randomised controlled trials to prove its value, and it is mostly used for some automatic jobs. Furthermore, the legal aspects and ethics of the usage of this type of technology need to be clarified more in the future. Because of its immense potential and essential need to use large quantities of data to train itself, AI is dependent on more efficient and easier ways of data connection, which is, nowadays, the first step to the inclusion of this type of technology in everyday practice. In the following paper, we have set out to summarise some of the most common applications of AI in contemporary RM, mainly focusing on its nonsurgical aspects. Figure 1 summarises AI usage in reproductive medicine.

## 2. Materials and Methods

This narrative review was performed for all available articles published as of September 2022 in PubMed. The search keywords used included “artificial intelligence and infertility,” “artificial intelligence and female infertility,” “artificial intelligence and male infertility,” “artificial intelligence and oocytes,” “artificial intelligence and embryos,” “in vitro fertilization and artificial intelligence,” “artificial intelligence and assisted reproductive technology”, “artificial intelligence and reproductive medicine”, “artificial intelligence and reproductive endocrinology”, “artificial intelligence ethics”, “legal/regulatory challenges”, and “human-centered AI”. All sources not specifically focused on the issue of an ethically and legally sustainable implementation of AI applications in reproductive care, and in healthcare as a whole, were left out.

## 3. Results

### 3.1. Artificial Intelligence and Female Infertility

Pregnancy rates after IVF treatment are approximately 30–70%, depending on the age of the female patient and the different protocol regimes used based on individual lists of parameters [41]. As mentioned before, female infertility may have several causes; hence, an adequate assessment is essential. The main steps in female infertility investigation are careful medical history, physical examination, endocrinologic assessment, ultrasound examination, hysterosalpingography, hysteroscopy, and laparoscopy [42].

Recently, many machine learning algorithms, including traditional logistic regression, support vector machines, decision trees, and random forests which have been presented in order to improve the ART success rate, using parameters such as age, body mass index, endometrial thickness, estradiol and progesterone level on the day of embryo transfer, type of infertility, good-quality embryo rate, and others [43].

Goyal et al. established the machine learning model to predict a successful live birth through 30 IVF clinical features [44], while others established six classification models to predict early pregnancy loss [45]. Many studies pointed out the random forest model as a platform in prediction [41]. Vogiatzi et al. constructed and validated an efficient ANN based on parameters with statistical correlations to live birth to predict clinical outcomes for patients undergoing ART [46].

The evaluation of ovarian reserve and endometrial receptivity using ultrasound (US) is very important for female fertility [47]. With regards to ovarian reserve follicular monitoring in the diagnosis of Polycystic Ovary Syndrome (PCOS), as well as prediction of oocyte quality and pregnancy outcomes, variables such as ovarian follicular diameter and volume, number of follicles, and ovarian stromal blood flow index were considered [48]. On the other hand, endometrial thickness and volume, endometrial morphology, and spiral arterial blood flow index are effective evaluation indicators [49]. There is a lot of ongoing research considering AI analysis of US images. These semiautonomous systems tend to save time and eliminate subjectiveness and inter- and intra-observer differences [50,51,52]. Hysteroscopy, as one of the main points in infertility investigation, is also being investigated within the scope of AI image analysis, with studies that successfully used the VGG NET 16 model to classify endometrial lesions [53].

Follicular fluid is very important for oocyte maturation and its quality. Some authors combined gonadotrophin levels, multivariate analysis and machine learning methods, and infrared spectra of follicle fluid to determine idiopathic female infertility [54]. Oocyte morphology assessment is of great importance for the successful fertilization rate, but it is also very useful during oocyte freezing for fertility preservation and in oocyte donation cycles. Adding oocyte morphology to AI may improve the precision of the algorithms. By including a total of 52 articles in this study, it has been shown that dark colour of the cytoplasm, homogeneous granularity of the cytoplasm, and ovoid shape of oocytes had no influence on treatment outcomes, but abnormalities such as refractile bodies, fragmented first polar body, dark zona pellucida, enlarged perivitelline space and debris were likely to affect the treatment outcome, whereas cytoplasmic vacuoles, centrally located cytoplasmic granularity, and clusters of smooth endoplasmic reticulum had negative impact on infertility treatment outcomes [55].

### 3.2. Embryo Transfer and Artificial Intelligence

It is of great importance to choose quality embryos for the transfer based on number of blastomeres, presence of nucleation and percentage of fragmentation. Pre-implantation genetic testing can be used in order to assess morphology, but with limited clinical utility [56]. Hence, newly described scoring systems such as AI have been developed, although their application and actual success rate still lacks clinically based evidence [57]. According to the Association for the Study of Reproductive Biology (ASEBIR) criteria and Gardner grading, inner cell mass, blastocyst expansion, and the trophectoderm are the best criteria for evaluating and selecting embryos on day five of development [58]. There is a tight connection between maternal and paternal Human Leukocyte Antigen [HLA] genes during embryo development and recurrent miscarriage, but no evident genetic markers have been identified. Mora-Sánchez and co. developed a methodology to analyze HLA haplotypes from couples with histories of either successful pregnancies or recurrent miscarriages in order to calculate the risk. This algorithm, called IMMATCH, is used to retrospectively predict recurrent miscarriage with an AUC = 0.71 (*p* = 0.0035) thanks to high-resolution typing and the use of linear algebra on peptide binding affinity data [59].

As mentioned earlier, the use of AI has been proposed as a viable solution for many of the present problems involving the empirical or subjective assessment of clinical and embryological decision points during the treatment of infertility, and embryo transfer (ET) is no exception. ET represents the last and most critical phase of the IVF procedure, and it is a crucial step, as the entire IVF cycle depends on the careful positioning of the embryos at the proper location—near the middle of the endometrial cavity. Therefore, achieving a live birth is the ultimate purpose of IVF, so the clinical decision making is centred on increasing a woman’s chance of getting pregnant [60]. ET is a stage composed of many variables, strategies, and techniques. During the previous century, and even nowadays, transferring several embryos was the most common strategy for a successful pregnancy [61]. However, not only does this method increase the chances of a viable pregnancy, but it also makes multiple pregnancies more likely, which is linked to higher rates of maternal and perinatal morbidity and mortality [62,63,64]. In light of this, the single embryo transfer (SET) approach has gained widespread acceptance as the only practical solution to resolve this issue and prevent multiple pregnancies in ART cycles. As a result, numerous countries have established regulations encouraging or requiring increased use of the SET approach [65,66,67,68,69]. Even so, to this day, reliable and valid ART outcome prediction is regarded as an unsolved issue, and no consensus yet exists among doctors as to treatment options and pregnancy probability estimation [60,70]. This is the point where AI gets into the picture.

Van Loendersloot et al. used the decision tree model to determine the cost-effectiveness of the different embryo transfer strategies in IVF in relation to female age. However, in their study, they also determined the more effective embryo transfer approach according to the age of the participants. This demonstrates how AI may address multiple problems at once and offer potential solutions. As expected, SET, followed by an additional frozen-thawed single embryo transfer if available and necessary, was dominant, less expensive, and more effective in patients under the age of 32, as shown in this study. On the contrary, in patients older than 32, double embryo transfer (DET) was shown to be more effective, but also more costly [71]. This opened the possibility of looking for a model that would predict the implantation outcome after an embryo transfer cycle, which could potentially increase the chances of a successful SET outcome in patients over the age of 32 and therefore improve the quality of life of the mother during and after the pregnancy, but also reduce the costs. This is exactly what Raef et al. aimed to do. They made a substantial contribution in the field of IVF using a significant ET data set with enough records to train a model using powerful machine learning prediction techniques. This data set included comprehensive and thorough aspects of patient demographics, embryo parameters, and cycle variables containing 82 features of IVF cycles. They discovered that among the six classification algorithms they used, random forest was the best classifier [70]. Recurrent implantation failure (RIF) is another big challenge for clinicians and a painful experience for couples that have experienced it. Shen et al. performed a study that provided a targeted and personalised treatment of RIF patients to help them achieve efficient and reliable pregnancy. They determined that among the four classification algorithms they used, the AdaBoost model obtained the best performance in the DET group, whereas the GBDT model proved to be the best in the SET group [72]. It is worth stressing that clinical judgment cannot be replaced by an AI decision-making system. It is a tool that provides suggestions and guides medical professionals in choosing the most individualised path to a successful pregnancy.

### 3.3. Artificial Intelligence and Male Infertility

By WHO estimations, the male factor contribution to infertility is somewhere around 50% of all causes of couple infertility. A lot of different medical conditions and states could facilitate male infertility, for instance, a variety of comorbidities such asliver failure, renal disease, chronic obstructive pulmonary disease, and multiple sclerosis. In addition, lifestyle factors, malignancies, drugs, hormonal disturbances, varicoceles, and recently investigated sperm DNA fragmentation can contribute to this state. Almost every medical condition that affects hormonal homeostasis, sexual function, and spermatogenesis could potentially contribute to male infertility. Nevertheless, 30% of all male infertility cases are still idiopathic [73]. Basic assessment of male infertility consists of taking a reproductive history and one or two semen sample analyses. Semen characteristics commonly analysed are sperm concentration, total sperm count, sperm motility, sperm vitality and sperm morphology. In a further investigation through hormonal evaluation, genetic testing and some imaging techniques could be obtained [3].

Numerous chemicals and physical agents produced by industrial or agricultural activities have been documented to have a significant negative impact on male reproductive function. These substances are frequently present in the environment and in certain occupational activities [74]. By assessing lifestyle and environmental factors, AI was used to predict the fertility rate and semen quality of male individuals. A data mining method using five different AI techniques (multilayer perception, decision tree, naive Bayes, support vector machine, and support vector machine + particle swarm optimisation) was applied to a data set that had environmental and lifestyle parameters and found that AI could be used to predict semen quality, analysing those parameters with good accuracy [75]. In a study performed a couple of years prior to the previous one, Gil et al. used three AI networks to analyse how environmental and/or lifestyle factors may have an impact on semen quality. The factors they evaluated included body mass index (BMI), alcohol intake, and cigarette smoking and/or exposure. They found that, out of the three AI methods they used, Multilayer Perceptron and Support Vector Machines showed the highest level of accuracy for sperm concentration (~86%) and for sperm motility (73–76%). Decision three, on the other hand, provided an excellent visual and illustrative approach, but still slightly lower accuracy [76]. Artificial neural networks used in another study based on questionnaire results obtained from male individuals also had 85.71% success in predicting the semen profile of the individual [77]. These studies prove that AI methods can be very useful to predict a person’s seminal profile based on environmental factors and lifestyle habits, and they can potentially lead to developing new preventive strategies and treatment methods for male infertility. There are different biochemical markers in seminal fluid that are associated with male infertility and could implicate semen quality (e.g., total protein content, fructose, glucosidase, zinc). In a study by Vicram and Rao Kamini, an ANN was constructed to predict those markers from categorised semen samples, with mean absolute error from −0.057 to 0.166 for different biomarkers [78]. The most advanced and broadly used investigation nowadays that relies on AI technology in RM is semen analysis (SA). AI is being used to perform this time-consuming job of SA, which is usually performed manually with a microscope [79]. SA performed by AI technology uses image and video sample assessment to categorise sperm characteristics. There are computer-aided sperm analysis (CASA) systems that can report motile percentage and kinematic parameters, as well as defining subpopulations of sperm cells [80].

A double blind prospective study, which compared two SA analysing systems (SQA-V GOLD and CASA CEROS) to the manual semen assessment, showed that CASA systems have advantages such as standardisation, speed, precision, reduced potential for human error, automated data recording, and less need for skilled professionals to run the systems. The problem with these systems lies in testing some atypical samples and the inability to perform an assessment of morphology abnormalities [81]. Riordon and McCallum trained ANNs with a true positive rate of 94% to classify sperm into WHO categories. They used freely available sperm data sets (HuSHeM and SCIAN). Furthermore, studies that investigated sperm motility applied machine learning to videos of sperm specimens and showed good and consistent prediction results [82]. Research studies that investigated machine learning spectrophotometry in SA found that it was more effective and reliable than the available spectrophotometry methods currently in use [83]. In addition, there is multimodal SA that uses data from SA video frames, patients’ serum levels of sex hormones, lifestyle habits, and semen parameters, so these data sets will be very practical for applying AI to investigate patterns that lie within [84]. Most importantly, digital home kits are being developed to allow SA in a domestic environment. These kits can be connected to a smartphone application and give results in real time, although these systems remain limited by reporting too few semen parameters. In the foreseeable future, more advanced systems accounting for more parameters should be developed, thus saving the time needed for an individual to go and bring specimens to the laboratories [85].

### 3.4. Artificial Intelligence and Idiopathic Infertility

Idiopathic or unexplained infertility affects 30% of couples worldwide. It is defined as an absence of a clear cause for a couple’s infertility and the female’s inability to become pregnant after at least 12 cycles of unprotected sexual activity, or after 6 cycles in the case of women over 35 years old whose standard evaluations are normal [86]. Environment and lifestyle factors have been pointed out as likely causes of idiopathic infertility, even if no definitive explanation has been found yet. Obesity and metabolic syndrome are known to have a negative impact on fertility in both men and women, even after using ART [87,88,89]. Bachelot et al. [90] conducted a study in which they set forth a very promising machine learning model that can stratify infertile/fertile couples on basis of their bioclinical signature, thus helping the management of couples with unexplained infertility. To our knowledge, this is the only study so far to use a couple-modelling approach instead of evaluating the parameters individually, since the cause of infertility is often multifactorial. Anthropometric assessment, antioxidative status, and metabolic status were among the variables used in this study. They used an unsupervised method, principal component analysis (PCA), and a supervised machine learning method, Orthogonal Partial Least Square-Discriminant Analysis (OPLS-DA). They also trained and assessed four additional machine learning models in order to confirm the discriminatory ability of the data, regardless of the model used—Support Vector Machine, Nearest Neighbours Classifier, Decision Tree and logistic regression using Python 3.8.2, Scikit-Learn library 0.22.2, Numpy library 1.18.1, and Pandas library 1.0.1. The suggested model may be used to manage couples with idiopathic infertility as part of routine care, but even though it is promising, prospective interventional studies are required to test the hypothesis this algorithm suggests and to certify such a model for clinical use [90].

### 3.5. Legal and Ethical Quandaries in AI-Based Healthcare

As has always been the case with any major scientific breakthrough throughout human history, innovative technologies with major potential to profoundly change vital aspects of our lives are likely to outpace the core values, ethics, and legal standards which govern society. That is even more true when reproductive medicine is involved, as was the case with practices such as heterologous fertilization [91]. The authors have therefore seen fit to briefly explore the approaches and actions currently being undertaken with regard to the legal, regulatory, and ethical governance of AI in healthcare. First, some AI methodologies are often regarded as “black-boxes”, in that they merely attempt to shed light on the relationship between input and output variables based in the training set. That implicates uncertainty regarding generalisation of the new data not included in previous data sets. In addition, when certain AI algorithms are used, there is no guarantee that an optimal solution can ultimately be achieved, and there are still few broadly shared guiding standards to determine how to tune parameters within algorithms [92]. When considering ethics of AI in medicine, the primary principle and priority of AI programming must always be safety. Programs must have transparency, credibility, auditability, and reliability, and they must be recoverable [93]. Machine learning processes need large amounts of data, and the rights to lawfully use such data are often unevenly regulated in different countries and regions, particularly in terms of the degree of de-identification of the patient’s identity [94]. AI implementation in RM still lacks randomised controlled trials, and there is growing awareness that AI models used in RM need to be interpretable and rigorously controlled before they can become mainstream in clinical practice [95]. The European Commission acknowledged such urgency and set out to launch a coordinated plan on AI with EU Member States (in addition to Switzerland and Norway) in December 2018 [96]. Technological shortcomings, e.g., a low degree of interoperability and standardisation among medical IT systems, also need to be dealt with. The ultimate purpose of such a blueprint is to foster AI development in Europe for the fundamental purpose of making the old continent the world-leading region for the development and implementation of highly innovative, but at the same time ethically sustainable, human-centred AI interventions—that is, conceiving and developing AI systems set to amplify and enhance, rather than supplant and displace, human capabilities [97]. A more recent and comprehensive noteworthy contribution from the Commission came in December 2019 with the release of the ethics guidelines for trustworthy artificial intelligence, in which the importance of AI in healthcare is highlighted in reference to Europe’s ageing population, as AI technologies and robotics have the potential to help caregivers provide the elderly with more timely and effective care, in addition to keeping patient conditions in check in real time [98]. Along similar lines, the first global report on Artificial Intelligence in health, which enunciates six pivotal guiding principles for its design and use, was issued by the World Health Organization on 28 June 2021 [99]. The WHO report puts forth a set of guidelines meant to reaffirm foundational precepts which emphasise, among other things, that human autonomy in the context of health care must be prioritised at all times, i.e., humans must keep control of health-care systems and medical decisions, whilst privacy and confidentiality are non-negotiable principles that must be upheld [26,100]. In addition, valid informed consent must be granted by patients through suitable legal frameworks for data protection. In order to achieve those goals, several factors will have to be accounted for in order to create a cultural and anthropological shift towards human-compatible AI by gaining a full understanding of how people engage with and trust AI systems [101]. Being able to explain and get through to people the operation of AI models and how AI systems operate is essential if we are to make the most out of the looming AI breakthrough set to affect, and hopefully improve, almost all aspects of our lives.

## 4. Conclusions

AI will bring a great innovation to the field of reproductive medicine and to healthcare as a whole through the improvement of treatment options for infertile patients, better planning of the procedures, and ultimately, higher ART success rates, thus reducing the costs of the treatment. It will be the optimal tool to help us predict clinical outcome based on the known initial parameters. Involving AI in everyday practice will take time, as well as concerted efforts to best harness the potential of risk-assessment systems and to reconfigure the way care is delivered. Ultimately, such a breakthrough will likely bring many advantages, such as the removal of the interfering factors: environmental and emotional factors or physical limitations. It will not replace human presence, but it will help with making the decision process in order to improve the final outcome and save time in the infertility treatment. The introduction of AI in ART procedures will revolutionise reproductive techniques, but it will definitely need a cautious and thoughtful approach, particularly when drafting legislative and regulatory frameworks solidly grounded in ethics precepts and core values, prioritising human dignity and upholding fundamental rights to privacy, data protection, and equality. Ultimately, such goals can only be achieved by preserving human control in order to make AI meet our needs, while at the same time operating transparently and achieving equitable outcomes. Such priorities are all the more essential when reproductive medicine is involved, since it also impacts the interest and well-being of unborn children. Guidelines, recommendations, and best practices based on widespread international consensus, at least among nations such as EU members, which share the same set of core values, are of utmost importance. To that end, all stakeholders need to make a concerted effort in order to make sure that human-centred AI, i.e., guided by human awareness and fundamental principles, will improve and enhance human skills and capabilities rather than replace them.

## Data Availability

Not applicable.
